# Diabetes mellitus and risk of breast cancer: a large-scale, prospective, population-based study

**DOI:** 10.1038/s41416-023-02345-4

**Published:** 2023-07-05

**Authors:** Fanxiu Xiong, Jingxuan Wang, Jovia L. Nierenberg, Erin L. Van Blarigan, Stacey A. Kenfield, June M. Chan, Gabriela Schmajuk, Chiung-Yu Huang, Rebecca E. Graff

**Affiliations:** 1 Department of Epidemiology and Biostatistics, University of California, San Francisco, San Francisco, CA USA; 2 Department of Urology, University of California, San Francisco, San Francisco, CA USA; 3 Helen Diller Family Comprehensive Cancer Center, University of California San Francisco, San Francisco, CA USA; 4 Division of Rheumatology, Department of Medicine, University of California, San Francisco, San Francisco, CA USA; 5 San Francisco Veterans Affairs Medical Center, San Francisco, CA USA

**Keywords:** Cancer epidemiology, Diabetes

## Abstract

**Background:**

The objective of this study was to evaluate associations of diabetes overall, type 1 diabetes (T1D), and type 2 diabetes (T2D) with breast cancer (BCa) risk.

**Methods:**

We included 250,312 women aged 40–69 years between 2006 and 2010 from the UK Biobank cohort. Adjusted hazard ratios (aHRs) and 95% confidence intervals (CIs) were calculated for associations of diabetes and its two major types with the time from enrollment to incident BCa.

**Results:**

We identified 8182 BCa cases during a median follow-up of 11.1 years. We found no overall association between diabetes and BCa risk (aHR = 1.02, 95% CI = 0.92–1.14). When accounting for diabetes subtype, women with T1D had a higher risk of BCa than women without diabetes (aHR = 1.52, 95% CI = 1.03–2.23). T2D was not associated with BCa risk overall (aHR = 1.00, 95% CI = 0.90–1.12). However, there was a significantly increased risk of BCa in the short time window after T2D diagnosis.

**Conclusions:**

Though we did not find an association between diabetes and BCa risk overall, an increased risk of BCa was observed shortly after T2D diagnosis. In addition, our data suggest that women with T1D may have an increased risk of BCa.

## Background

Diabetes mellitus is a growing epidemic of global proportions. It is estimated that in 2019, 463 million adults aged 20–79 years were living with diabetes, and the number is likely to grow substantially in future decades [1]. Diabetes occurs through two different primary disease processes. Type 2 diabetes mellitus (T2D) accounts for roughly 90% of diabetes cases worldwide [1]. It is characterized by decreased hepatic and extrahepatic insulin sensitivities and/or impaired insulin release. Type 1 diabetes mellitus (T1D) is characterized by autoimmune destruction of insulin-producing pancreatic ß cells that results in deficient insulin production. Diabetes-associated metabolic disorders, hormonal antecedents, and its treatments could plausibly affect the risk of cancer [2].

Breast cancer (BCa) is the most prevalent cancer in women and the most commonly diagnosed cancer globally, with 2.26 million new cases in 2020 [3]. Diabetes has been proposed to promote BCa initiation through several biological pathways, including alterations of the hyperinsulinemia/insulin-like growth factor (IGF) axis, hyperglycemia, fat-induced inflammation, and changes in sex hormone levels [2, 4–6]. Insulin resistance and hyperinsulinemia are less prominent in T1D than T2D [7], but mechanisms underlying a possible association with T1D specifically have not been extensively considered.

Meta-analyses and large cohort studies have suggested an increased risk of BCa in diabetic individuals [8–10]. In the largest meta-analysis, which included 40 studies [8], women with diabetes had a significantly increased risk of BCa relative to non-diabetic women (summary relative risk = 1.27, 95% confidence interval (CI): 1.16–1.39). In secondary analyses, only T2D was positively associated with BCa risk; the relationship for T1D was null. In addition, diabetes was only associated with the risk of postmenopausal, and not premenopausal, BCa. Though the meta-analysis was significant overall, so too was the heterogeneity among studies. Differences in epidemiological design could not readily explain the differences, and many studies did not adjust for body mass index (BMI) or other potential confounders. Furthermore, most studies were primarily composed of participants with T2D and/or did not distinguish between the two major types of diabetes. Because of differences in etiology, drug therapies, age at onset, and body composition (wherein individuals with T1D are generally leaner than those with T2D), findings for T2D should not be directly applied to T1D.

The heterogeneity of findings and limited research on T1D support the need for further investigation of the relationship between diabetes and BCa. We thus examined associations of diabetes overall, T1D, and T2D with risk of incident BCa using data from the population-based UK Biobank cohort.

## Methods

### Study population

The UK Biobank is a prospective cohort of 502,647 adults aged 40–69 years when they were recruited between 2006 and 2010. The UK Biobank has approval from the North West Multi-centre Research Ethics Committee. At recruitment, all participants provided written informed consent, completed baseline questionnaires and interviews, supplied biospecimens, and underwent physical exams. Since baseline, participants have been followed via linkage to the National Health Service (NHS) Central Register. For these analyses, we excluded participants who: self-reported as male and/or demonstrated male genetic sex (*n* = 229,255); self-reported cancer but did not have cancer registry records or had neoplasms of uncertain or unknown behavior (*n* = 1262); had a history of any cancer prior to baseline or a cancer diagnosis without a corresponding diagnosis date (*n* = 15,348); had a mastectomy (NHS procedure codes: OPCS version 3—383, 384, 385; OPCS version 4—B27) before baseline or mastectomy without corresponding surgery date (*n* = 6469); or had a recorded death date prior to baseline (*n* = 1). The remaining 250,312 women comprised our study population.

### Exposure

Prevalent and incident diabetes cases were identified using the 10th Revision of the International Classification of Diseases (ICD-10) and/or self-report [11]. ICD-10 codes for insulin-dependent diabetes mellitus (IDDM) and non-insulin-dependent diabetes mellitus (NIDDM) were E10 and E11, respectively. Self-reported diabetes (any diabetes, T1D, or T2D) and the corresponding date that a doctor first diagnosed diabetes were collected during the baseline interview and subsequent assessment center visits based on a standardized questionnaire with good internal validity. Only 8% of diabetes cases, all of which were prevalent, were identified by self-report alone. Based on our meta-analysis of 70 studies investigating the relationship between diabetes and BCa risk [12], the method of diabetes ascertainment does not seem to substantially influence results.

We determined that IDDM and NIDDM do not adequately distinguish T1D and T2D; many participants diagnosed with IDDM had been previously diagnosed with NIDDM or had self-reported T2D that had become insulin dependent. As suggested by previous studies [7, 13], we therefore reclassified participants as having T1D if their earliest age at diabetes diagnosis, whether based on ICD-10 codes or self-reported, was 30 years or younger. All remaining participants were classified as having T2D. Diabetes was considered prevalent if the date of diagnosis was prior to study entry and incident if the diabetes was diagnosed during follow-up. Those who reported only having diabetes during pregnancy (i.e., gestational diabetes) were included in the non-diabetic group.

For secondary analyses, we also identified baseline self-reported history of anti-diabetic medication use, including metformin (yes, no) and insulin (yes, no), among participants with T2D.

### Outcome

The outcome of interest was first diagnosis of BCa. Incident cancer cases were identified through linkage to national cancer registries, which is considered to be the gold standard approach in the UK [14]. BCa was determined by an ICD-9 code of 174 or an ICD-10 code of C50. Follow-up data for the UK Biobank cohort were available until June 30, 2020 for England, January 31, 2019 for Wales, and 30 June 2018 for Scotland, at which points the relevant cancer registries last captured a BCa diagnosis.

### Covariates

Multivariable models were adjusted for a set of confounding and BCa risk factors determined a priori, namely age at baseline assessment (continuous), self-reported race (white, non-white), Townsend Deprivation Index (TDI; quintiles; higher scores indicate greater levels of deprivation or socioeconomic disadvantage) [15], BMI (<25.0, 25.0−< 30.0, ≥30.0 kg/m^2^), physical activity (<20, 20− < 40, 40− < 60, ≥60 metabolic equivalent hours (METh)/week, unknown), smoking status and intensity (never, former, current <15 cigarettes/day, current ≥15 cigarettes/day, current intensity unknown), alcohol consumption (never, special occasions or 1–3 times per month, 1–4 times per week, daily or almost daily), educational level (higher, secondary, vocational, other), family history of BCa in a mother or sister (yes, no), ever mammography (yes, no), ever use of oral contraceptives (OCs; yes, no), ever use of hormone replacement therapy (HRT; yes, no), age at menarche (<12, 12–13, 14–15, ≥16 years), menopausal status (pre, post; age 50 was used as a proxy for menopausal status for the <5% of women for whom data for this field were missing); parity (nulliparous, 1, 2, ≥3 children), and age at first live birth (nulliparous, <20 years, 20– < 29, ≥30). All covariables were measured at baseline.

To explore possible underlying mechanisms of any relationship between diabetes and BCa, a set of biomarkers was identified for additional multivariable models. Per the protocol of the UK Biobank Biomarkers Project, blood samples were collected at the assessment centers, and serum concentrations of a range of key biomarkers were measured using a phased analysis approach [16]. For these analyses, we utilized testosterone (nmol/L), IGF-1 (nmol/L), sex hormone binding globulin (SHBG; nmol/L), C-reactive protein (CRP; mg/L), and haemoglobin A1c (HbA1c; mmol/mol). Estrogen was not considered due to substantial missingness.

### Statistical analysis

Participants were followed from study enrollment until the first of the following events: BCa diagnosis, diagnosis of a different cancer, non-cancer-related radical mastectomy, death, or the last date at which follow-up was considered complete. The non-BCa endpoints were considered censoring events. Diabetes was treated as a time-varying exposure, wherein women with incident diabetes contributed person-years to the no diabetes group before their diagnosis of diabetes and contributed person-years to the diabetes group thereafter. Kaplan–Meier curves were used to summarize survival for women with diabetes (any diabetes, T1D, or T2D) at baseline compared to those without diabetes at baseline. Differences in survival by diabetes status and types were assessed using log-rank tests.

Cox proportional hazards models were employed to estimate hazard ratios (HRs) and 95% CIs for associations between diabetes and risk of BCa. Length of follow-up was the time scale in all models. Multivariable models were adjusted for the aforementioned set of covariables. In secondary analyses, we implemented multivariable models further categorizing women with T2D by metformin use (no, yes), insulin use (no, yes), and diabetes duration (<5, 5− < 10, 10− < 15, ≥15 years). Diabetes duration was calculated from the date of diabetes diagnosis to the date of BCa diagnosis or censoring. We tested the proportional hazards assumption using the Schoenfeld residual test and did not find evidence of a violation.

We assessed effect modification of associations between diabetes and BCa by the following variables, all measured at baseline: age at assessment (40− < 50, 50−< 60, ≥60 years), BMI (<25, 25− < 30, ≥30 kg/m^2^), ever had a mammogram (yes, no), ever HRT use (yes, no), age at menarche (<12, ≥12 years), menopausal status (pre, post), and parity (nulliparous, parous). Interactions between diabetes and potential effect modifiers were tested by entering cross-product terms into the multivariable models. In additional analyses, the aforementioned pre-determined biomarkers were entered in the multivariable models individually and in combination. The potential role of these serologic factors was evaluated with likelihood ratio tests.

Finally, we conducted several sensitivity analyses for the associations of diabetes and its two major types with BCa: (1) limiting exposure to incident diabetes; (2) redefining T1D as diagnosis prior to age 20 years; (3) redefining T2D as diagnosis after age 40 years; (4) excluding participants diagnosed with BCa within one year of study entry or diabetes diagnosis; (5) using Fine and Gray competing risks analysis to account for malignant cancers other than breast and death; (6) using age as the underlying time scale; (7) and including an interaction term between diabetes (or diabetes subtype) and BMI.

All statistical tests were two-sided with *P* < 0.05 considered to be statistically significant. Analyses were conducted using R statistical software, version 4.0.2.

## Results

Our study included 575 women with T1D at baseline, 7891 women with T2D at baseline, 6821 women who developed T2D over a median follow-up of 11.1 years (interquartile range: 10.4–11.8 years), and 235,025 women who had never been diagnosed with either type of diabetes by the end of follow-up (Table 1). Compared to non-diabetic participants, women with either type of diabetes were more likely to be non-White, have higher TDIs, and never have used OCs. Women with T1D were also less likely to have ever had a mammogram, be postmenopausal, and be parous at baseline. Women with prevalent versus incident T2D had similar baseline characteristics, excepting the substantially less frequent use of metformin or insulin for the latter women. Relative to both non-diabetic women and women with T1D, women with T2D consumed less alcohol and had less education. They were also more likely to have higher BMI, have undergone mammography, have ever used HRT, and be postmenopausal than non-diabetic women. Regarding serum markers, women with T1D demonstrated the highest levels of SHBG and HbA1c, while the participants with T2D had the lowest levels of SHBG but the highest serum concentrations of CRP. In all, 76.3% of the participants with T1D and 53.8% of the participants with prevalent T2D had elevated HbA1c (HbA1c ≥47.5 mmol/mol). Among women who developed incident diabetes during follow-up, 13.6% had elevated HbA1c at baseline versus 0.33% in those who did not develop diabetes.

**Table 1:** Baseline characteristics of 250,312 female UK Biobank participants by diabetes status and subtypes.

Characteristic	No diabetes	T1D^a^	T2D	
	*n* = 235,025	*n* = 575	Prevalent	Incident
			*n* = 7891	*n* = 6821
Age at assessment (years), mean (SD)	56.0 (8.0)	53.4 (8.1)	59.8 (6.9)	58.6 (7.5)
White, *n* (%)	222,017 (94.5)	514 (89.4)	6652 (84.3)	5877 (86.2)
TDI, mean (SD)	−1.40 (3.00)	−0.54 (3.30)	−0.27 (3.38)	−0.35 (3.38)
BMI (kg/m^2^), mean (SD)	26.8 (4.9)	28.0 (5.6)	32.6 (6.6)	32.1 (6.2)
Physical activity (METh/week), median (IQR)	37.0 (48.8)	36.0 (48.0)	35.8 (48.7)	35.5 (51.5)
Smoking status and intensity, *n* (%)				
Never	140,743 (59.9)	347 (60.3)	4477 (56.7)	3674 (53.9)
Former	72,557 (30.9)	166 (28.9)	2643 (33.5)	2160 (31.7)
Current, <15 cig/day	7668 (3.3)	19 (3.3)	207 (2.6)	261 (3.8)
Current, ≥15 cig/day	7707 (3.3)	22 (3.8)	353 (4.5)	492 (7.2)
Current, intensity unknown	5134 (2.2)	12 (2.1)	127 (1.6)	165 (2.4)
Alcohol consumption, *n* (%)				
Never	20,561 (8.7)	79 (13.7)	1805 (22.9)	1294 (19.0)
Special occasions or 1–3 times per month	63,873 (27.2)	167 (29.0)	3314 (42.0)	2669 (39.1)
1–4 times per week	110,852 (47.2)	229 (39.8)	2156 (27.3)	2276 (33.4)
Daily or almost daily	39,140 (16.7)	96 (16.7)	566 (7.2)	542 (7.9)
Education, *n* (%)				
Higher	87,843 (37.4)	227 (39.5)	2151 (27.3)	1646 (24.1)
Secondary	95,838 (40.8)	241 (41.9)	2767 (35.1)	2451 (35.9)
Vocational	10,228 (4.4)	15 (2.6)	429 (5.4)	443 (6.5)
Other	38,995 (16.6)	83 (14.4)	2458 (31.1)	2159 (31.7)
Family history of BCa, *n* (%)	15,237 (6.5)	28 (4.9)	384 (4.9)	332 (4.9)
Ever had a mammogram, *n* (%)	183,289 (78.0)	382 (66.4)	6968 (88.3)	5818 (85.3)
Ever OCs use, *n* (%)	191,405 (81.4)	412 (71.7)	5568 (70.6)	4980 (73.0)
Ever HRT use, *n* (%)	86,894 (37.0)	182 (31.7)	3531 (44.7)	3164 (46.4)
Age at menarche (years), mean (SD)	12.6 (2.7)	12.4 (3.5)	12.3 (3.0)	12.4 (3.0)
Menopause, *n* (%)	169,913 (72.3)	354 (61.6)	6975 (88.4)	5749 (84.3)
Parity, *n* (%)				
Nulliparous	44,229 (18.8)	179 (31.1)	1337 (16.9)	1049 (15.4)
1 child	31,333 (13.3)	111 (19.3)	1026 (13.0)	889 (13.0)
2 children	103,377 (44.0)	182 (31.7)	2986 (37.8)	2627 (38.5)
3 or more children	55,428 (23.6)	99 (17.2)	2480 (31.4)	2212 (32.4)
Age at first birth (years), mean (SD)^b^	26.1 (5.2)	26.2 (5.4)	24.3 (4.9)	24.0 (4.8)
Metformin, *n* (%)	N/A	N/A	4368 (55.4)	124 (1.8)
Insulin, *n* (%)	N/A	N/A	1428 (18.1)	22 (0.3)
Testosterone (nmol/L), median (IQR)	1.02 (0.65)	1.16 (0.87)	0.98 (0.66)	1.03 (0.71)
SHBG (nmol/L), mean (SD)	63.2 (30.8)	72.9 (39.4)	41.4 (25.4)	42.4 (25.5)
IGF-1 (nmol/L), mean (SD)	21.2 (5.7)	18.2 (5.8)	19.2 (6.5)	19.3 (6.2)
HbA1c (mmol/mol), median (IQR)	34.9 (4.7)	60.1 (17.8)	49.8 (14.8)	41.1 (6.8)
CRP (mg/L), median (IQR)	1.30 (2.16)	1.92 (3.35)	2.49 (4.03)	3.35 (4.82)

*BCa* breast cancer, *BMI* body mass index, *cig* cigarettes, *CRP* C-reactive protein, *HbA1c* haemoglobin A1c, *HRT* hormone replacement therapy, *IGF* insulin-like growth factor, *IQR* interquartile range, *METh* metabolic equivalent of task hours, *OC* oral contraceptive, *SD* standard deviation, *SHBG* sex hormone-binding globulin, *T1D* type 1 diabetes, *T2D* type 2 diabetes, *TDI* Townsend deprivation index.

^a^All type 1 diabetes cases were identified prior to baseline assessment.

^b^Mean age at first birth was calculated among parous women.

**Fig. 1:**
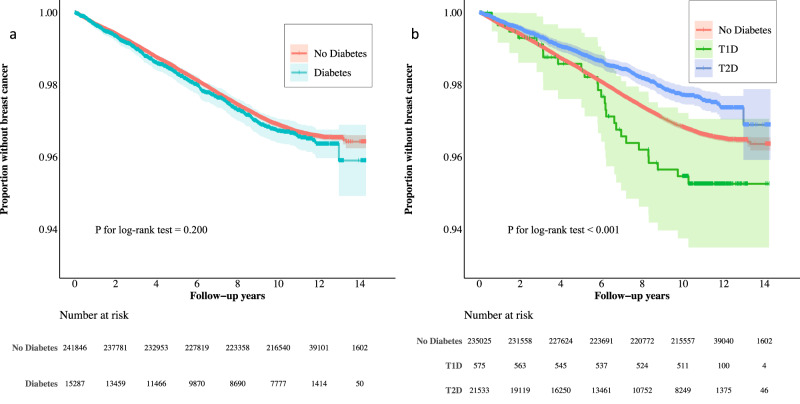
Kaplan–Meier curves of breast cancer incidence for 250,312 female UK Biobank participants. Shading represents 95% confidence intervals. **a** Breast cancer incidence by diabetes status. **b** Breast cancer incidence by diabetes subtype. T1D type 1 diabetes, T2D type 2 diabetes.

Women without diabetes contributed nearly 2.6 million person-years of follow-up versus 119,481 person-years among those with diabetes (Table 2). There were 7745 incident BCa diagnoses in the former group and 383 in the latter. Based on Kaplan–Meier curves, women with diabetes demonstrated similar times to developing BCa as the non-diabetic group (*P* = 0.20; Fig. 1). The incidence rates of BCa per 100,000 person-years were 300.5 for non-diabetic women and 320.6 for diabetic women. Multivariable Cox models also indicated no overall association between diabetes and BCa risk (adjusted hazard ratio (aHR) = 1.02, 95% CI = 0.92–1.14). Similarly, having T2D was not associated with BCa (aHR = 1.00, 95% CI = 0.90–1.12). Women with T1D, however, had a higher incidence rate of BCa compared to women without diabetes (428.0 vs. 300.5 cases/100,000 person-years), and multivariable models suggested a positive association between history of T1D and developing BCa (aHR = 1.52, 95% CI = 1.03–2.23).

**Table 2:** Associations of diabetes, its subtypes, medications for type 2 diabetes, and type 2 diabetes duration with breast cancer risk among 250,312 female participants in the UK Biobank.

Diabetes status	*N* Diabetes	PYs	N BCa	Incidence/100,000 PYs	Age-adjusted HR (95% CI)	Multivariable-adjusted HR (95% CI)^b^
No diabetes	235,025	2,577,777	7,745	300.5	1.00 (ref)	1.00 (ref)
All diabetes	15,287	119,481	383	320.6	1.03 (0.93–1.14)	1.02 (0.92–1.14)
T1D	575	6075	26	428.0	1.50 (1.02–2.21)	1.52 (1.03–2.23)
T2D	14,712	113,406	357	314.8	1.01 (0.90–1.12)	1.00 (0.90–1.12)
Metformin use^a^						
No	10,220	81820	211	257.9	1.03 (0.90–1.18)	1.03 (0.89–1.18)
Yes	4492	46167	146	316.2	0.95 (0.81–1.12)	0.93 (0.79–1.10)
Insulin use^a^						
No	13,262	113,084	308	272.4	1.00 (0.89–1.12)	1.00 (0.89–1.13)
Yes	1450	14,903	49	328.8	1.00 (0.76–1.33)	0.96 (0.72–1.28)
T2D duration, years^a^						
<5	4253	10,480	172	1641.2	3.95 (3.25–4.80)	3.94 (3.23–4.80)
5– < 10	2675	18,173	129	709.8	1.91 (1.58–2.31)	1.88 (1.55–2.28)
10– < 15	3469	37,092	83	223.8	0.68 (0.54–0.84)	0.66 (0.53–0.82)
≥15	4315	47,662	63	132.2	0.40 (0.31–0.51)	0.39 (0.30–0.50)
*P* for trend					<0.001	<0.001

*BCa* breast cancer, *CI* confidence interval, *PYs* person-years, *HR* hazard ratio, *T1D* type 1 diabetes, *T2D* type 2 diabetes.

^a^Model was tested by comparing sub-population with type 2 diabetes to women without diabetes.

^b^Adjusted for age at baseline, self-reported race, Townsend deprivation index, body mass index, physical activity, smoking status and intensity, alcohol consumption, educational level, family history of breast cancer in biological relatives, ever had a mammogram, ever use of oral contraceptives, ever use of hormone replacement therapy, age at menarche, menopausal status, parity, and age at first live birth.

Relative to women without diabetes, neither baseline metformin nor insulin use among women with T2D was associated with BCa risk (Table 2). However, increasing duration of T2D was significantly associated with reduced BCa risk (*P* for trend <0.001). The risk of BCa was highest in the first 5 years after T2D diagnosis (aHR = 3.94, 95% CI = 3.23–4.80). With increasing time more than 5 years after T2D diagnosis, BCa risk consistently declined. The direction of the association between T2D and BCa reversed after 10 years since T2D diagnosis (aHR = 0.66, 95% CI = 0.53–0.82) and was most strongly inverse among participants diagnosed with T2D more than 15 years prior to BCa diagnosis (aHR = 0.39, 95% CI = 0.30–0.50).

**Fig. 2:**
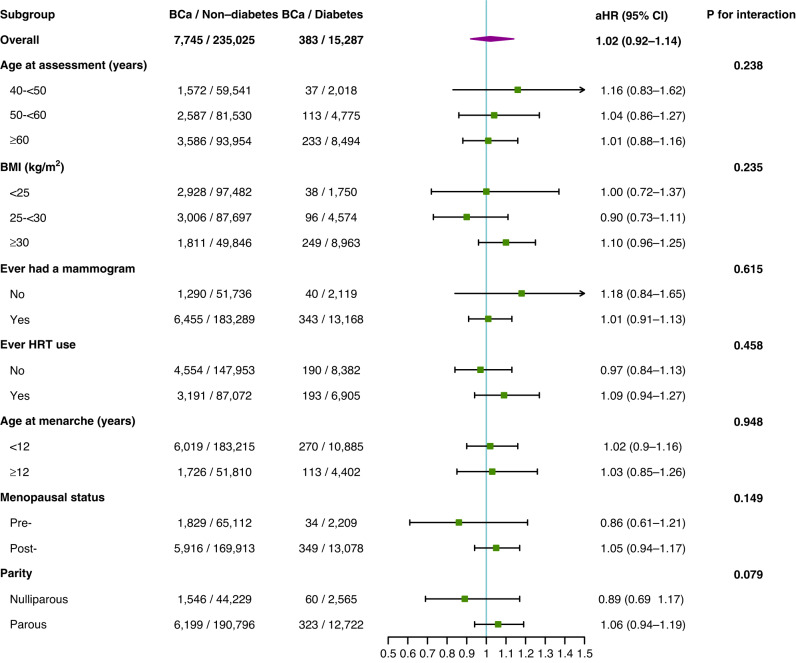
Associations between diabetes and breast cancer risk in the UK Biobank cohort across subgroups defined by age at assessment, BMI, ever had a mammogram, ever HRT use, age at menarche, menopausal status, and parity. BMI body mass index, HRT hormone replacement therapy, kg/m2 kilograms per square meter.

Associations for diabetes overall and T2D with BCa generally remained null in subgroups defined by age at assessment, BMI, ever had a mammogram, ever HRT use, age at menarche, menopausal status, and parity (Fig. 2 and Supplementary Table 1). However, T1D was linked to elevated BCa risk among women aged 60 and older (aHR = 2.40, 95% CI = 1.00–5.78), who had ever had a mammogram (aHR = 1.56, 95% CI = 1.01–2.43), who ever used HRT (aHR = 3.01, 95% CI = 1.35–6.73), who were postmenopausal (aHR = 1.65, 95% CI = 1.05–2.59), and who were parous (aHR = 1.68, 95% CI = 1.08–2.61). We did not observe statistically significant interactions between any of these variables and overall diabetes, T1D, or T2D (Fig. 2 and Supplementary Table 1).

Including serum markers in the multivariable models did not materially change the results (Supplementary Table 2). Results from sensitivity analyses restricting analysis to incident diabetes, redefining T1D and T2D, and excluding BCa cases that occurred within 1 year of baseline or diabetes diagnosis, and from models including an interaction term between diabetes or its subtypes with BMI were similar to the main findings (Supplementary Table 3). However, the results from Fine and Gray models accounting for the competing risks of malignant cancer other than breast and death and Cox proportional hazards models using age as the underlying time scale showed significantly reduced BCa risk for women with T2D (Supplementary Table 3). The cause-specific hazard function for competing events revealed that women with diabetes were likely to have other types of malignant cancer or death than women without diabetes.

## Discussion

In this large prospective cohort of women in the UK with a median 11.1 years of follow-up, having diabetes was not generally associated with the risk of developing BCa. However, T1D was associated with an increased risk of BCa, and our data showed that women with T2D may be more likely to be diagnosed with BCa during the first decade after diabetes diagnosis.

Previous meta-analyses and large cohorts have indicated a modest increased risk of BCa among postmenopausal women with diabetes [7, 8, 10, 17]. However, studies have demonstrated substantial heterogeneity, and many have not adjusted for BMI—which is strongly associated with diabetes and a well-established risk factor for postmenopausal BCa—or other possible confounding factors. The overall null results of our study are consistent with results from a Women’s Health Initiative (WHI) multicenter study [18, 19], a study within the British Columbia Linked Health Databases (BCLHD) [20], and the most recently published findings from the Sister Study [21]. Consistent with the summary estimate, our null findings did not vary by age categories, menopausal status, BMI, or other BCa risk factors.

The few previous studies that have investigated BCa occurrence among persons with T1D have reported heterogeneous results [7, 13, 22–24]. Single-register studies have reported no association [7, 13, 22, 24], but a pooled analysis of five nationwide diabetes registers indicated a statistically significantly decreased risk of BCa in individuals with T1D [23]. These studies, however, did not adjust for BCa-specific risk factors, such as reproductive characteristics. Though we were able to adjust for such factors, our finding of a positive association between T1D and BCa risk should be interpreted with caution. Only 26 incident BCa cases were diagnosed among 576 women with T1D. In addition, all T1D was prevalent at baseline and diagnosed at a young age. Because our dataset did not include follow-up in the early window following T1D onset, we were unable to estimate BCa incidence in the short period following T1D diagnosis.

In our study, the hazard of BCa in participants with T2D showed a clear temporal trend, with an initial spike in the first 5 years following diabetes diagnosis and reduced BCa risk after 10 years from the diabetes index date. It is unlikely that BCa diagnosed shortly after diabetes onset is due to diabetes-related carcinogenesis. Rather, women with newly diagnosed diabetes likely have more frequent contact with health care providers and therefore have more BCa screening opportunities [25, 26]. Indeed, at baseline, a higher proportion of women with than without T2D in our cohort reported having ever undergone BCa screening. Failure to account for detection bias occurring in the early window after diabetes diagnosis may lead to overestimation of the association between diabetes and BCa risk.

More than 10 years after T2D diagnosis, BCa risk was statistically significantly inverse relative to individuals without T2D. T2D was also significantly associated with reduced BCa risk when using age as the underlying time scale in the Cox proportional hazards model. It is challenging to disentangle whether the pattern is solely due to the cumulative effects of T2D or some combination of disease duration, anti-diabetic medication use [21], and lifestyle modification. Survival bias could play a role if factors that improve survival of diabetic patients are associated with lower BCa risk. It is also possible that frequent censoring from competing causes in diabetic individuals results in fewer BCa cases over time. Certainly there is considerable evidence that diabetes is linked to an increased risk of several types of cancer [27] and independently increases mortality related to cardiovascular disease, renal disease, and cancer by 1.3–3.0 times [28]. Our Fine and Gray models indicated a significantly reduced BCa risk in women with diabetes. However, results from an a posteriori analysis of diabetes duration and BCa risk using Fine and Gray models indicated that the inverse association only presented with more than 10 years since diabetes diagnosis (data not shown). It is also plausible that individuals with longstanding chronic disease are less likely to receive routine cancer screening compared to the general population. To determine the likelihood that biology explains the inverse relationship between long-term diabetes and BCa, it would be important to rule out such biases.

In our analyses, metformin and insulin use among individuals with T2D were not associated with BCa risk compared to those without T2D. Biguanide metformin is the first-line medication for individuals with T2D, and in some situations, it has been repurposed as an anti-cancer drug [29]. Several mechanisms through which metformin could have anti-cancer properties have been proposed, most of which involve AMP kinase activation that leads to inhibition of the insulin/IGF-1 pathway, mammalian target of rapamycin (mTOR) pathway, and human epidermal growth factor receptor type 2 (HER-2) expression [29–31]. Multiple studies have suggested reduced BCa incidence and cancer-related mortality in individuals using metformin [32–34]. However, these findings may have been confounded by indication [2], since individuals prescribed metformin generally have been only recently diagnosed with diabetes and are in better health. Insulin and its analogs are hormonal treatments for glycemic control. Some studies have suggested that insulin increases the risk of BCa [35, 36], but other studies have been inconclusive [37, 38]. Studies of insulin and BCa may also experience confounding by indication, given that insulin is more often used among participants with a longer duration of T2D and in those with more comorbid conditions. Our results should be interpreted with caution because we assessed metformin and insulin use at baseline only, lacked information on medication dosage, frequency, and duration, and did not evaluate other types of anti-diabetic medications.

Strengths of this study include its prospective design, ICD coding of diabetes and BCa, linkage to cancer registries, available data for a relatively complete set of confounding factors, and the ability to explore anti-diabetic medications and possible biological links between diabetes and BCa. However, there are several limitations that deserve mention. First, competing risks are a concern since women with diabetes are less likely to develop BCa than other types of malignant cancer or death in the long term. Second, the incidence of BCa during follow-up—301 cases per 100,000 person-years—is higher than the estimated 210 cases per 100,000 females per year in the UK [39]. The difference may be attributable to the age distribution of the UK Biobank cohort compared to the general population. Third, the majority of the study participants were white. Generalizations of our results must thus be made cautiously. Fourth, it is essential to explore the relationships between diabetes and molecular subtypes of BCa, which have diverse etiologies and behaviors. Unfortunately, tumor molecular subtypes are currently unavailable in the UK Biobank. Fifth, data from the UK Biobank may include some measurement error, and we were unable to validate our exposure and outcome measurements. Finally, we had limited statistical power to examine the effect modifiers in the context of the association between T1D and BCa.

In conclusion, we did not observe associations between diabetes and BCa among women in the UK Biobank overall. However, our data suggested that women with T1D or recently diagnosed with T2D may have an increased BCa risk. Larger studies among other races/ethnicities are warranted to further investigate the relationship between T1D and BCa risk.

## Supplementary information

Supplementary Table 1

Supplementary Table 2

Supplementary Table 3

## Data Availability

Bona fide researchers can apply to use the open-access UK Biobank dataset by registering and applying at http://ukbiobank.ac.uk/register-apply/.

## Code availability

Code is available from the authors upon request.
