# Supplementary material for: Diabetes mellitus and risk of breast cancer: a large-scale, prospective, population-based study
Source: Br J Cancer. 2023 Jul 5;129(4):648–55. doi: 10.1038/s41416-023-02345-4 (PMC10421865; doi:10.1038/s41416-023-02345-4)
Supplement: Supplementary file 1 — Supplementary Table 1 [file 41416_2023_2345_MOESM1_ESM.docx]

**Supplementary Table 1.** Associations of diabetes and its subtypes with breast cancer risk among 250,312 female participants in the UK Biobank across subgroups defined by age at assessment, menopausal status, body mass index, ever use of hormone replacement therapy, ever had a mammogram, parity, and age at menarche.

|  |  | **Type 1 diabetes** | | | |  | **Type 2 diabetes** | | | |  |
| --- | --- | --- | --- | --- | --- | --- | --- | --- | --- | --- | --- |
| **Subgroup** |  | **N BCa** | **N T1D** | **aHR (95% CI)^a^** | **P for interaction** |  | **N BCa** | **N T2D** | **aHR (95% CI)^a^** | **P for interaction** |  |
| Overall |  | 26 | 575 | 1.52 (1.03-2.23) |  |  | 357 | 14712 | 1.00 (0.90-1.12) |  |  |
| Age at assessment (years) | | | |  | 0.128 |  |  |  |  | 0.165 |  |
| 40-<50 |  | 6 | 208 | 1.09 (0.49-2.44) |  |  | 31 | 1810 | 1.17 (0.81-1.69) |  |  |
| 50-<60 |  | 8 | 200 | 1.33 (0.66-2.65) |  |  | 105 | 4575 | 1.03 (0.84-1.26) |  |  |
| ≥60 |  | 12 | 167 | 1.95 (1.11-3.45) |  |  | 221 | 8327 | 0.98 (0.85-1.13) |  |  |
| BMI (kg/m^2^) | |  |  |  | 0.728 |  |  |  |  | 0.134 |  |
| <25 |  | 8 | 186 | 1.43 (0.71-2.86) |  |  | 30 | 1564 | 0.92 (0.64-1.32) |  |  |
| 25-<30 |  | 9 | 212 | 1.37 (0.71-2.65) |  |  | 87 | 4362 | 0.87 (0.70-1.08) |  |  |
| ≥30 |  | 9 | 177 | 1.72 (0.89-3.32) |  |  | 240 | 8786 | 1.08 (0.94-1.24) |  |  |
| Ever had a mammogram | | |  |  | 0.609 |  |  |  |  | 0.637 |  |
| No |  | 6 | 193 | 1.25 (0.56-2.78) |  |  | 34 | 1926 | 1.17 (0.81-1.68) |  |  |
| Yes |  | 20 | 382 | 1.56 (1.01-2.43) |  |  | 323 | 12786 | 0.99 (0.88-1.11) |  |  |
| Ever HRT use | |  |  |  | 0.071 |  |  |  |  | 0.684 |  |
| No |  | 12 | 393 | 1.08 (0.61-1.91) |  |  | 178 | 7989 | 0.96 (0.82-1.13) |  |  |
| Yes |  | 14 | 182 | 2.21 (1.30-3.73) |  |  | 179 | 6723 | 1.05 (0.90-1.22) |  |  |
| Age at menarche (years) | | |  |  | 0.565 |  |  |  |  | 0.855 |  |
| <12 |  | 18 | 425 | 1.38 (0.87-2.20) |  |  | 252 | 10460 | 1.00 (0.88-1.14) |  |  |
| ≥12 |  | 8 | 150 | 1.76 (0.88-3.52) |  |  | 105 | 4252 | 1.00 (0.81-1.23) |  |  |
| Menopausal status | | |  |  | 0.416 |  |  |  |  | 0.118 |  |
| Pre- |  | 7 | 221 | 1.16 (0.55-2.43) |  |  | 27 | 1988 | 0.80 (0.54-1.18) |  |  |
| Post- |  | 19 | 354 | 1.65 (1.05-2.59) |  |  | 330 | 12724 | 1.02 (0.91-1.15) |  |  |

**Supplementary Table 1 (Cont.).** Risk of breast cancer associated with diabetes and its subtypes among 250,312 female participants in the UK Biobank across subgroups defined by age at assessment, menopausal status, body mass index, ever use of hormone replacement therapy, ever had a mammogram, parity, and age at menarche.

|  |  | **Type 1 diabetes** | | | |  | **Type 2 diabetes** | | | |  |
| --- | --- | --- | --- | --- | --- | --- | --- | --- | --- | --- | --- |
| **Subgroup** |  | **N BCa** | **N T1D** | **aHR (95% CI)^a^** | **P for interaction** |  | **N BCa** | **N T2D** | **aHR (95% CI)^a^** | **P for interaction** |  |
| Parity |  |  |  |  | 0.168 |  |  |  |  | 0.156 |  |
| Nulliparous | | 6 | 179 | 1.07 (0.48-2.38) |  |  | 54 | 2386 | 0.88 (0.66-1.16) |  |  |
| Parous |  | 20 | 396 | 1.68 (1.08-2.61) |  |  | 303 | 12326 | 1.03 (0.91-1.16) |  |  |
|  |  |  |  |  |  |  |  |  |  |  |  |
| Abbreviations: aHR, adjusted hazard ratio; BCa, breast cancer; BMI, body mass index; CI, confidence interval; HRT, hormone replacement therapy; T1D, type 1 diabetes; T2D, type 2 diabetes. | | | | | | | | | | | |
| ^a^Adjusted for age at baseline, self-reported race, Townsend deprivation index, body mass index, physical activity, smoking status and intensity, alcohol consumption, educational level, family history of breast cancer in biological relatives, ever had a mammogram, ever use of oral contraceptives, ever use of hormone replacement therapy, age at menarche, menopausal status, parity, and age at first live birth (except in the analyses stratified by parity). | | | | | | | | | | | |
