# Supplementary material for: Diabetes mellitus and risk of breast cancer: a large-scale, prospective, population-based study
Source: Br J Cancer. 2023 Jul 5;129(4):648–55. doi: 10.1038/s41416-023-02345-4 (PMC10421865; doi:10.1038/s41416-023-02345-4)
Supplement: Supplementary file 2 — Supplementary Table 2 [file 41416_2023_2345_MOESM2_ESM.docx]

**Supplementary Table 2.** Associations of diabetes and its subtypes with breast cancer risk among 250,312 female participants in the UK Biobank, when additionally adjusting for serum markers in the models.

|  |  | **All Diabetes** | |  | **Type 1 diabetes** | |  | **Type 2 diabetes** | |  |
| --- | --- | --- | --- | --- | --- | --- | --- | --- | --- | --- |
| **Inclusion of:** |  | **aHR (95% CI)^a^** | **P for LRT** |  | **aHR (95% CI)^a^** | **P for LRT** |  | **aHR (95% CI)^a^** | **P for LRT** |  |
| No serum markers |  | 1.03 (0.92-1.14) |  |  | 1.52 (1.03-2.23) |  |  | 1.00 (0.90-1.12) |  |  |
| Testosterone |  | 1.04 (0.92-1.17) | 1.000 |  | 1.28 (0.81-2.03) | 0.000 |  | 1.03 (0.91-1.16) | <0.001 |  |
| IGF-1 |  | 1.05 (0.94-1.17) | <0.001 |  | 1.50 (0.99-2.29) | 0.000 |  | 1.03 (0.92-1.15) | <0.001 |  |
| SHBG |  | 1.01 (0.90-1.14) | <0.001 |  | 1.43 (0.91-2.25) | 0.000 |  | 0.99 (0.88-1.12) | <0.001 |  |
| CRP |  | 1.04 (0.93-1.16) | 0.234 |  | 1.43 (0.94-2.17) | 0.257 |  | 1.02 (0.91-1.14) | 0.314 |  |
| HbA1c |  | 1.05 (0.92-1.19) | 0.315 |  | 1.57 (1.01-2.43) | 0.444 |  | 1.03 (0.90-1.18) | 0.213 |  |
| Five serum markers |  | 1.07 (0.92-1.25) | <0.001 |  | 1.49 (0.87-2.56) | 0.000 |  | 1.05 (0.90-1.23) | <0.001 |  |
|  |  |  |  |  |  |  |  |  |  |  |
| Abbreviations: aHR, adjusted hazard ratio; CI, confidence interval; CRP: C-reactive protein; HbA1c, hemoglobin A1c; IGF-1, insulin-like growth factor-1; LRT, likelihood ratio test; SHBG, sex hormone-binding globulin. | | | | | | | | | | |
| ^a^Adjusted for age at baseline, self-reported race, Townsend deprivation index, body mass index, physical activity, smoking status and intensity, alcohol consumption, educational level, family history of breast cancer in biological relatives, ever had a mammogram, ever use of oral contraceptives, ever use of hormone replacement therapy, age at menarche, menopausal status, parity, and age at first live birth. | | | | | | | | | | |
