# Supplementary material for: Diabetes mellitus and risk of breast cancer: a large-scale, prospective, population-based study
Source: Br J Cancer. 2023 Jul 5;129(4):648–55. doi: 10.1038/s41416-023-02345-4 (PMC10421865; doi:10.1038/s41416-023-02345-4)
Supplement: Supplementary file 3 — Supplementary Table 3 [file 41416_2023_2345_MOESM3_ESM.docx]

**Supplementary Table 3.** Sensitivity analyses for associations of diabetes and its two major subtypes with breast cancer risk among 250,312 female participants in the UK Biobank.

| **Diabetes status** | **N Diabetes** | **PYs** | **N BCa** | **Incidence /100,000 PYs** | **aHR (95% CI)^a^** |
| --- | --- | --- | --- | --- | --- |
| Incident diabetes | 6,821 | 30,727 | 90 | 292.9 | 1.01 (0.82-1.25) |
| T1D prior to age 20 years | 299 | 3,163 | 14 | 442.6 | 1.53 (0.90-2.58) |
| T2D after age 40 years | 14,193 | 107,894 | 340 | 315.1 | 0.99 (0.89-1.11) |
| Excluding BCa within 1 year of baseline or diabetes diagnosis | | | | | |
| All diabetes | 15,188 | 118,987 | 346 | 290.8 | 0.96 (0.86-1.08) |
| T1D | 570 | 6,084 | 24 | 394.5 | 1.53 (1.03-2.29) |
| T2D | 14,618 | 112,903 | 322 | 285.2 | 0.94 (0.83-1.05) |
| Fine and Gray analysis |  |  |  |  |  |
| All diabetes | 15,287 | 119,481 | 383 | 320.6 | 0.76 (0.68-0.85) |
| T1D | 575 | 6,075 | 26 | 428.0 | 1.48 (0.98-2.23) |
| T2D | 14,712 | 113,406 | 357 | 314.8 | 0.73 (0.65-0.82) |
| Age as underlying time scale |  |  |  |  |  |
| All diabetes | 15,287 | 134,362 | 383 | 285.1 | 0.45 (0.40-0.50) |
| T1D | 575 | 6,075 | 26 | 428.0 | 1.45 (0.99-2.14) |
| T2D | 14,712 | 128,286 | 357 | 278.3 | 0.42 (0.37-0.47) |
| Including an interaction term between diabetes and BMI | | | | | |
| All diabetes | 15,287 | 119,481 | 383 | 320.6 | 0.98 (0.71-1.35) |
| T1D | 575 | 6,075 | 26 | 428.0 | 1.45 (0.72-2.91) |
| T2D | 14,712 | 113,406 | 357 | 314.8 | 0.91 (0.63-1.30) |
|  |  |  |  |  |  |
| Abbreviations: aHR, adjusted hazard ratio; BCa, breast cancer; BMI, body mass index; CI, confidence interval; PY, person-year; T1D, type 1 diabetes; T2D, type 2 diabetes. | | | | | |
| ^a^Adjusted for age at baseline, self-reported race, Townsend deprivation index, body mass index, physical activity, smoking status and intensity, alcohol consumption, educational level, family history of breast cancer in biological relatives, ever had a mammogram, ever use of oral contraceptives, ever use of hormone replacement therapy, age at menarche, menopausal status, parity, and age at first live birth. | | | | | |
